# A Pilot Study to Investigate Peripheral Low-Level Chronic LPS Injection as a Model of Neutrophil Activation in the Periphery and Brain in Mice

**DOI:** 10.3390/ijms25105357

**Published:** 2024-05-14

**Authors:** Michelle Aries, Makayla Cook, Tiffany Hensley-McBain

**Affiliations:** 1 McLaughlin Research Institute, Great Falls, MT 59405, USA; michelle.aries@mclaughlinresearch.org (M.A.);; 2 Department of Basic Sciences, Touro College of Osteopathic Medicine Montana, Great Falls, MT 59405, USA

**Keywords:** chronic inflammation, immune activation, neutrophil, extracellular traps, low-level LPS, IP injection, peripheral, brain, flow cytometry, microscopy, C57BL/6J, mice, timepoint optimization

## Abstract

Lipopolysaccharide-induced (LPS) inflammation is used as model to understand the role of inflammation in brain diseases. However, no studies have assessed the ability of peripheral low-level chronic LPS to induce neutrophil activation in the periphery and brain. Subclinical levels of LPS were injected intraperitoneally into mice to investigate its impacts on neutrophil frequency and activation. Neutrophil activation, as measured by CD11b expression, was higher in LPS-injected mice compared to saline-injected mice after 4 weeks but not 8 weeks of injections. Neutrophil frequency and activation increased in the periphery 4–12 h and 4–8 h after the fourth and final injection, respectively. Increased levels of G-CSF, TNFa, IL-6, and CXCL2 were observed in the plasma along with increased neutrophil elastase, a marker of neutrophil extracellular traps, peaking 4 h following the final injection. Neutrophil activation was increased in the brain of LPS-injected mice when compared to saline-injected mice 4–8 h after the final injection. These results indicate that subclinical levels of peripheral LPS induces neutrophil activation in the periphery and brain. This model of chronic low-level systemic inflammation could be used to understand how neutrophils may act as mediators of the periphery–brain axis of inflammation with age and/or in mouse models of neurodegenerative or neuroinflammatory disease.

## 1. Introduction

Neuroinflammation and immune activation are widely accepted as significant contributing factors to the pathophysiology of Alzheimer’s disease (AD), Parkinson’s disease (PD), multiple sclerosis (MS), amyotrophic lateral sclerosis (ALS), Huntington’s disease (HD), and other neurodegenerative diseases [1,2,3]. Moreover, neuroinflammation and immune activation are present before the onset of symptoms [4,5]. An increase in proinflammatory cytokines, such as tumor necrosis factor-alpha (TNFα) and interleukin-1b (IL-1b), and caustic molecules released during inflammation, such as nitric oxide (NO) and myeloperoxidase (MPO), have been associated with a worse prognosis across multiple neurodegenerative diseases [2,6]. Neuroinflammatory markers such as these, along with immune cells, including neutrophils and microglia, are potential therapeutic targets for multiple neurodegenerative diseases.

Neutrophils are of therapeutic interest because they are the most abundant circulating leukocyte in humans and have been shown in both human and mouse models to be associated with a worse prognosis in neurodegenerative diseases [7,8,9]. Genes involved in neutrophil activation and adhesion have been identified in areas with blood–brain barrier (BBB) disruption and associated with disease progression [10,11,12,13]. Furthermore, humanized AD mouse model studies have shown an increase in short-term spatial memory and a decrease in capillary blood flow stalling when targeting neutrophil adhesion or accumulation [9,14,15].

Neutrophils are essential to fight invaders and clear debris [16]. Neutrophils mainly contain pathogens through phagocytosis, degranulation, and the formation of neutrophil extracellular traps (NETs). While neutrophils are important in fighting infection and repairing tissues, they can also cause damage to tissues through their release of antimicrobial peptides, enzymes meant to degrade the extracellular matrix, and reactive oxygen species [17,18,19]. Neutrophils are not commonly found in a healthy brain because of their exclusion by the BBB [16,20]. However, the BBB is disrupted in neurogenerative diseases such as AD [17,21] and PD [22]. Although neutrophils have been documented in the brain in several neuroinflammatory conditions and diseases, including stroke, multiple sclerosis, and AD, the role of peripheral inflammation in neutrophil infiltration and activation in the brain is unclear [14,17,21,23,24]. Administration of high levels of lipopolysaccharide (LPS) into the periphery as a model of sepsis results in neutrophil infiltration into the brain [25,26,27]. However, the level of inflammation that is induced in sepsis models is not representative of the chronic low-level inflammation that occurs with age and likely contributes to age-related neurological disease. Previous studies have also demonstrated that neutrophils adhere to the vasculature, infiltrate the brain parenchyma, and can return to the blood stream after interaction with microglia following acute systemic LPS exposure in mice and rats [28,29]. However, no studies have investigated chronic peripheral inflammation and subsequent neutrophil activation in the brain and periphery. With this pilot study, we demonstrate that low-level chronic LPS in the periphery induces neutrophil activation in the periphery of the brain. The LPS exposure model outlined in this pilot study can be further explored in future studies in mice of different genetic backgrounds to investigate the role of neutrophils in neuroinflammation and related brain disorders. This will be specifically useful to understand how peripheral mediators and genetic risk factors for disease may alter neutrophil responses to peripheral stimuli and if neutrophils are mechanistic drivers of the subsequent neuroinflammation and neurodegeneration.

## 2. Results

### 2.1. Comparing 4-Week vs. 8-Week Durations of Chronic LPS Exposure to Induce Peripheral Neutrophil Activation

No studies investigating chronic LPS-induced neuroinflammation and its potential cognitive consequences conducted to date have investigated the induction of neutrophil activation in the periphery or brain. As such, we sought to investigate the administration of 0.5 mg/kg of LPS once per week as a potential model of neutrophil activation in the brain. This LPS exposure represents the threshold of physiological changes in mice, and can be used to model low-level chronic inflammation, as may occur in persons with microbiome alterations or frequent GI or respiratory infections common in elderly individuals [30]. This dose has also been demonstrated to impact amyloid pathology and levels of inflammatory cytokines in AD mouse models [31,32]. It was therefore selected based on an interest to use this model in future studies to investigate neutrophilic contributions to AD.

**Figure 1:**
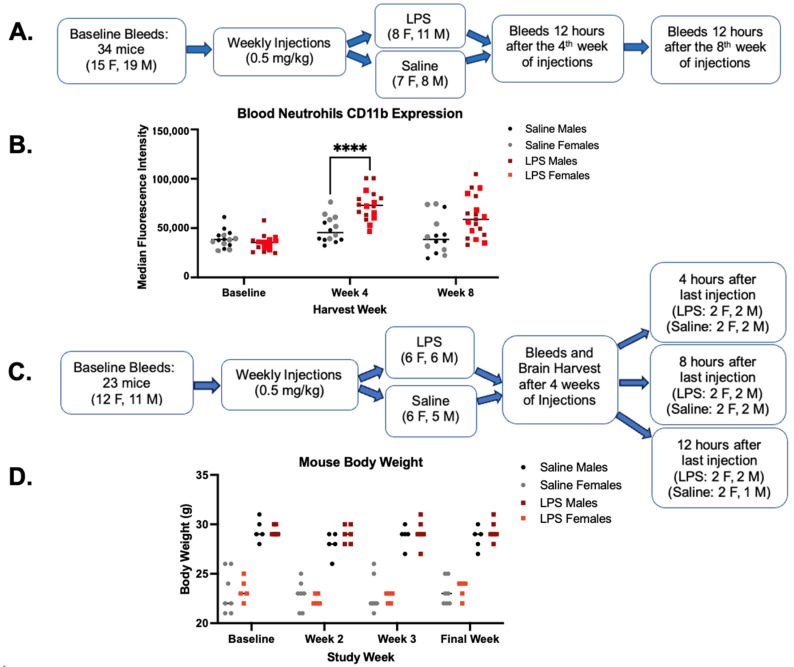
Study design iterations. (**A**) Schematic overview of the preliminary LPS pilot study design to compare 4 vs. 8 weeks of LPS or saline injections. (**B**) Median CD11b expression as measured by flow cytometry on peripheral neutrophils from the initial 8-week pilot described in (**A**) in LPS-injected mice and saline-injected mice demonstrating increased neutrophil activation. (**C**) Schematic overview of study design for the next iteration of the study. Blood and brain tissue were collected after 4 weeks of injections. Neutrophil frequency, activation, and soluble markers were measured 4 h, 8 h, and 12 h after the last injection. (**D**) Body weight throughout the duration of study is outlined in schematic C. Statistical significance was assessed between the LPS and saline groups across timepoints by repeated measures 2-way ANOVA followed by post hoc assessments within each timepoint using Tukey’s multiple comparisons test. Multiplicity adjusted P values are represented as **** *p* < 0.0001.

We first sought to determine whether 4 or 8 weeks of chronic LPS injections was optimal to induce neutrophil activation in the periphery (Figure 1A). In this first iteration of the pilot study, male and female mice were injected IP with 0.5 mg/kg of LPS or saline once per week for 8 weeks, and blood was collected at baseline (pre-injection), 12 h after the 4th injection, and 12 h after the 8th injection (Figure 1A). Following injections, neutrophil activation was assessed via flow cytometry (Figure S1). CD11b expression in neutrophils was higher in LPS-injected mice compared to saline-injected mice after 4 weeks of injections (*p* < 0.0001) but not after 8 weeks of injections (Figure 1B). This could be due to increased tolerance, reflected as a slightly lower response in LPS-injected mice, or due to increased inflammation in a subset of saline-injected mice at 8 weeks (Figure 1B). Of note, neutrophil activation was non-significantly increased in the saline-injected group at both 4 weeks and 8 weeks, likely representing inflammation associated with injection and handling. Based on these data, the next group of mice was injected for 4 weeks to investigate the timing of neutrophil activation in the brain following the final LPS injection (Figure 1C). Blood was drawn at baseline, and blood and brain tissue were collected 4 h, 8 h, or 12 h after the 4th and final injection (Figure 1C). As expected with this level of LPS, there were no significant changes in body weight in either the saline- or LPS-injected mice throughout the experiment (Figure 1D).

### 2.2. Peripheral Neutrophil Activation following Low-Level Chronic LPS

**Figure 2:**
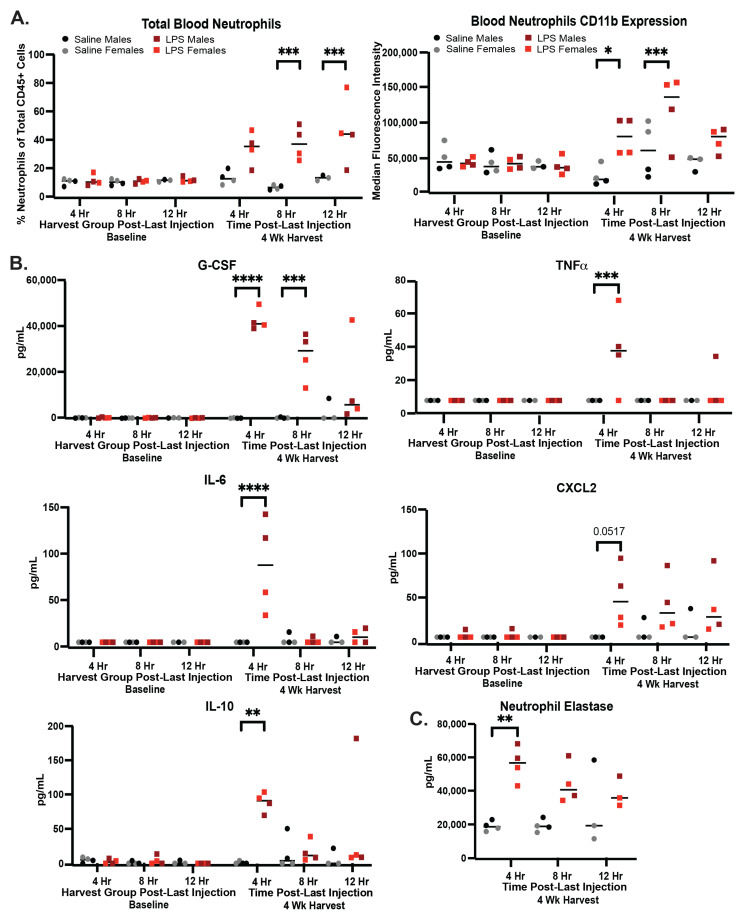
Increased peripheral neutrophil activation following 4 weeks of chronic low-level LPS injections. (**A**) Neutrophil frequency in peripheral blood and median CD11b expression on peripheral blood neutrophils as measured by flow cytometry in mice receiving LPS (red and dark red squares) or saline (grey and black circles). Neutrophils are defined as live, CD45+, CD11b+, Ly6G+ cells (Figure S1). (**B**) Increased cytokines and chemokines in plasma as measured by multiplex bead array (LegendPlex) in mice receiving LPS (red and dark red squares) or saline (grey and black circles). (**C**) Increased neutrophil elastase in plasma as measured by ELISA in mice receiving LPS (red and dark red squares) or saline (grey and black circles). A and B were measured in submandibular blood, and C was measured in additional cardiac blood collected at time of euthanasia. Statistical significance was assessed between the LPS and saline groups across timepoints by 2-way ANOVA followed by post hoc assessments within each timepoint using Tukey’s multiple comparisons test. Multiplicity adjusted *p* values are represented as * *p* < 0.05, ** *p* < 0.01, *** *p* < 0.001, **** *p* < 0.0001.

Following 4 weeks of IP LPS injections, blood neutrophil frequency and activation were assessed via flow cytometry (Figure S1). No significant increases in blood neutrophil frequency or activation were observed in the control mice receiving saline. Increases in neutrophil frequencies were observed 4 h after the final LPS injection and reached statistical significance 8 and 12 h following LPS injection, *p* < 0.001 (Figure 2A). Of note, the lack of a statistically significant increase compared to saline-injected mice at 4 h may be partially due to the slight increase in neutrophils in the saline-injected controls, potentially reflecting inflammation caused by handling and injection. Neutrophil CD11b expression increased 4 h after the final LPS injection (*p* < 0.05), peaking at 8 h following LPS injection (*p* < 0.001) (Figure 2A). Peripheral cytokines and chemokines with the ability to regulate neutrophil activation, maturation, and recruitment were measured in plasma via a multiplex bead array. Of the 12 total analytes measured, 5 showed a statistically significant change between the LPS and saline groups. G-CSF, TNFa, IL-6, and CXCL2 increased 4 h after LPS injection, with G-CSF remaining elevated 8 h post LPS administration (*p* < 0.001) (Figure 2B). The anti-inflammatory mediator IL-10 was also increased 4 h following the final LPS injection (*p* < 0.01), suggesting an early potential compensatory response following LPS injection [4,5]. Finally, neutrophil elastase (NE), a marker of neutrophil extracellular trap release [6], was measured via ELISA in plasma. NE was significantly increased 4 h following LPS injection (*p* < 0.01) and remained elevated but not significantly elevated 8 and 12 h following LPS injection (Figure 2C). Taken together, these data suggest that soluble mediators of neutrophil activation rapidly increase by 4 h after IP LPS injection, followed by an increase in neutrophil frequency and neutrophil activation in the blood that peaked 8 h after LPS administration. Soluble mediators of neutrophil responses and neutrophil activation begin to resolve 8–12 h following LPS. However, neutrophil frequency remains elevated 12 h post-LPS. 

### 2.3. Brain Neutrophil Activation following Low-Level Chronic LPS

**Figure 3:**
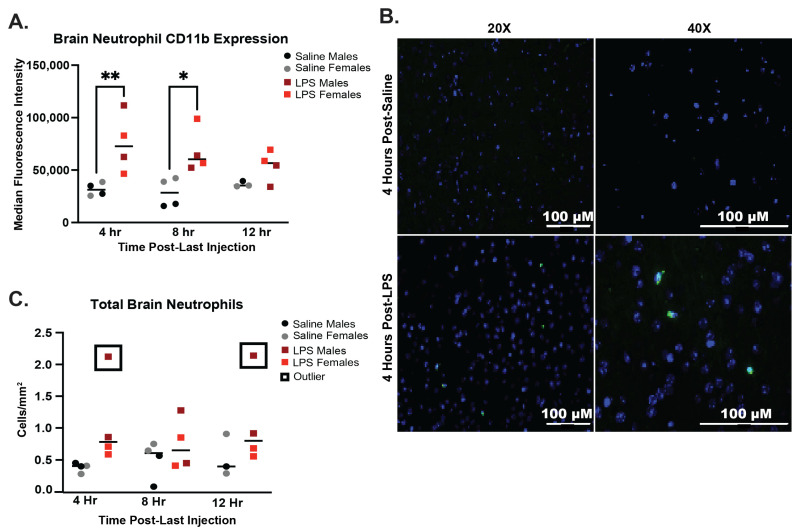
Infiltration of neutrophils into the brain and activation of brain neutrophils following 4 weeks of chronic low-level LPS injections. (**A**) Neutrophils in the brain were assessed by flow cytometry (Figure S1). Neutrophils were defined as CD45+CD11b+Ly6G+ cells, and activation was assessed via CD11b expression. (**B**) Example images of neutrophils stained with MPO-specific antibody (green) and DAPI (blue) in the visual area of the cortex of saline- and LPS-injected mice. (**C**) Neutrophils counted and averaged via microscopy in two sagittal brain sections and normalized by area in saline- and LPS-injected mice. Outliers were identified by ROUT method and indicated by a black box. Outliers were not included in statistical assessments. Statistical significance was assessed across timepoints by 2-way ANOVA followed by post hoc assessments within each timepoint using Tukey’s multiple comparisons test. Multiplicity adjusted *p* values are represented as * *p* < 0.05, ** *p* < 0.01.

Brain neutrophil activation, as measured by CD11b expression, was significantly increased in mice receiving LPS 4 h (*p* < 0.01) and 8 h after injection (*p* < 0.05), which corresponds to what was observed for peripheral neutrophils (Figure 3A). We assessed total neutrophils in the brain via microscopy. Brain neutrophils were identified in sagittal sections via myeloperoxidase (MPO) staining using an antibody that has been validated to stain for neutrophils in mouse and human brain tissue (Figure 3B) [17]. A previous study has demonstrated that MPO-staining in mouse and human brain tissue is neutrophil-specific [17]. In addition, we validated the MPO staining of neutrophils by demonstrating that anti-MPO-positive cells have multilobed nuclei consistent with neutrophils and that MPO colocalizes with the neutrophil marker S100A8 (Figure S3). Neutrophils were counted and averaged across two entire sagittal sections and were variably elevated in the brain across timepoints (Figure 3C). Increased neutrophils in the brain were not statistically significant after accounting for statistical outliers in the 4 h and 8 h harvest groups. In addition, neutrophil frequencies were higher in the 8 h and 12 h groups of saline-injected mice compared to the 4 h groups. Neutrophil infiltration into specific brain regions was also variable, with influx into the cortex observed at 4 h and 12 h but not 8 h after the final LPS injection (Figure S2). Given the limited size and variability in neutrophil frequency, potential inferences about total neutrophils in the brain and in specific brain regions are limited with these data. However, different mice in the 4 h and 12 h LPS groups demonstrated over a 2-fold higher number of neutrophils in the brain, particularly in the cortex, suggesting that this may be an area of future study. Due to the potential for neutrophil-microglia crosstalk, we additionally assessed microglial frequencies in the brain in this model and found no statistically significant increase in microglia (Figure S4). Taken together these data indicate that neutrophils demonstrate increased activation in brain tissue within 4 h following peripheral low-level LPS injection. 

## 3. Discussion

LPS is a component of the outer membrane of Gram-negative bacteria that binds to toll-like receptor 4 (TLR-4) on immune cells. It is commonly used to induce inflammation in the periphery and brain. However, studies differ in their dose, frequency, and route of administration [3]. Most studies have investigated acute exposure to LPS, administering LPS intraperitoneally (IP) daily for 5–7 days at doses of 0.25–1 mg/kg. Chronic exposure studies have demonstrated microglial and astrocyte activation and memory impairment with similar doses administered IP once or twice weekly for 4–6 weeks [33]. Despite LPS as a common model for peripheral immune activation and subsequent neuroinflammation, no studies have investigated the role of neutrophils in mediating the periphery–brain axis of inflammation induced by chronic low levels of LPS. Neutrophils in the periphery and vasculature could contribute to neuroinflammation and neurodegeneration in multiple ways, including their role in blood flow stalling and their direct release of proinflammatory cytokines and factors that disrupt the BBB [7,34]. In addition, peripheral inflammation could result in increased neutrophil adhesion molecules that result in their extravasation into the brain and release of granules and NET components that may directly damage tissue. Neutrophils in the brain could impact the surrounding tissue in multiple ways. Their release of ROS, NETs, and cytokines can activate microglia, and their release of extracellular histones can induce neuronal apoptosis [16,20,35]. The matrix metalloproteinases (MMPs) and proteases released by neutrophils during degranulation or the generation of NETs results in breakdown of the extracellular matrix and neuronal damage [16,20]. 

With this study, we sought to determine if low levels of peripheral LPS result in neutrophilic contributions to neuroinflammation. Models of various neurodegenerative diseases have demonstrated that chronic low-level LPS administered IP results in neuroinflammation, as evidenced by induced glial activation, cognitive dysfunction, cerebrovascular leakiness, and inflammatory cytokines in the brain [33]. Chronic peripheral LPS in these models also induced proteinopathy, with increased amyloid deposition in AD models and TDP-43 aggregation in ALS models [33]. The role of neutrophils in mediating neurodegeneration in response to low levels of chronic LPS in the periphery has never been examined. Here, we report that chronic IP LPS injections for 4 weeks induces increased neutrophils activation in the periphery and brain in mice. In our preliminary assessments, we found that neutrophil activation in the periphery was higher in LPS-injected mice compared with saline-injected mice after 4 weeks but not after 8 weeks of injections, therefore limiting our brain investigations to 4 weeks. Elevated neutrophil frequencies were observed through 12 h post-LPS in the periphery. Maintained elevated frequencies suggest that neutrophils released from the bone marrow are not yet undergoing homeostatic apoptosis by 12 h post-LPS [36]. Future studies should investigate the role of fewer and additional injections on neutrophils in the brain and the impact on the temporal nature of the response. 

There are multiple mechanisms for how peripheral LPS may result in increased neutrophil activation in the brain. It has been demonstrated that LPS signaling through the TLR-4 receptor increases CD11b expression on neutrophils, and the binding of CD11b/CD18 (Mac 1) to ICAM-1 mediates adhesion and transmigration, which are necessary for extravasation into tissues [37]. We observed increased CD11b expression on peripheral and brain neutrophils following chronic low-level LPS. Therefore, it is possible that LPS increases neutrophil adhesion and extravasation into the brain through increased CD11b expression. Importantly, blood neutrophil CD11b expression is increased in persons with AD and correlates with disease severity, making it a relevant marker of neutrophil activation in models of neuroinflammation and neurodegeneration [38]. A previous study demonstrated that neutrophils enter the brain in 5xFAD mice, a mouse model for AD, through binding of LFA-1 to integrins, and LFA-1-dependent neutrophil recruitment has also been observed in a model of LPS-induced lung inflammation [9]. We did not measure LFA-1 (CD11a/CD18) on the surface of neutrophils in this study, nor did we measure receptor expression levels (e.g., ICAM-1). However, this model can be used to further understand how peripheral inflammation may result in neutrophil migration into the brain and decipher which adhesion molecules are involved. In addition, peripheral inflammation can contribute to BBB dysfunction, thus allowing for inflammatory cytokines and peripheral immune cells to more easily traffic into the brain and activate microglia that perpetuate neuroinflammation and continued BBB dysfunction [39]. Neutrophils have been found near areas of BBB dysfunction in AD mouse models, but whether the BBB is a cause or a consequence of neutrophil invasion remains to be determined [7]. 

Increased CD11b mediates phagocytosis and oxidative burst in neutrophils and is increased on peripheral neutrophils in AD [38,40], potentially due to increased TNFa [41,42,43]. In our study, TNFa increased 4 h following the final LPS injection but resolved by 8 h post-LPS, and CD11b expression was sustained through 8 h post-LPS in both the periphery and brain but resolved by 12 h post-LPS. This suggests that weekly LPS injections do not result in sustained elevations of TNFa and neutrophil activation at this very low dose. It will be interesting in future studies to determine how sustained exposure to subclinical inflammatory stimuli may alter these responses in comparison to intermittent stimuli, which we tested here. However, previous studies have demonstrated that exposure to this level of LPS early in life resulted in cognitive impairments 10 months later in mice [44,45], suggesting that damage caused by leukocytes that infiltrate temporarily may result in sustained impairment or may synergize later with age to contribute to degeneration. We did not investigate neurodegeneration or cognitive impairment in this pilot study. However, this will be an important area of future study. Finally, CD11b expression on neutrophils increases with age, so CD11b expression and neutrophil activation in mice receiving low-level chronic LPS should be further examined at different ages to determine how age impacts this model [46].

Overall, the data provided here are evidence that chronic, low-level LPS administered in the periphery increases neutrophil activation in the periphery and brain. While we did not study functionality of neutrophils in this study, the observed increase in NE is suggestive of increased NET release, which has been observed as a peripheral marker of neurodegeneration and neuroinflammation [35]. NET release in the brain is known to contribute to damage, so future studies should investigate NETs in the brain following low levels of peripheral LPS exposure. Of note, neutrophils may also have suppressive functions and contribute to the resolution of inflammation [7]. Studies have demonstrated that LPS may induce regulatory T cells that in turn result in the production of IL-10 by neutrophils, and this interaction is mediated by CD11b [47]. However, two observations suggest that this is not the main mechanism observed in our study: (1) IL-10 peaks at 4 h post-LPS, while neutrophils remain elevated through 8–12 h, suggesting that, at least at later timepoints, neutrophils are not producing high levels of IL-10. (2) Neutrophils that produce IL-10 were demonstrated to have decreased CD11b expression in a prior study [47], and neutrophils in this model upregulated CD11b expression following LPS injection. Future studies should perform functional assays to assess NET release, phagocytic ability, and ability to suppress T cell function to fully elucidate how neutrophils contribute to inflammation in this model. In addition, a detailed assessment of neutrophil dynamics as they relate to activation and frequency following LPS-injection will be of high importance. The relationship between CD11b expression and neutrophil frequency could be assessed by investigating neutrophil lifespan ex vivo and the expression of activation markers in conjunction with apoptosis markers, such as active Caspase-3, as we have previously investigated in human neutrophils [36].

This study has several limitations, some of which have already been discussed. First, this is a small pilot study, and although we included both male and female mice, we are underpowered to analyze them separately. Sex differences should be examined in the future to understand how this model may be applied to investigate sexual dimorphism in neuroinflammation. Given the sample size used herein, these results may not be generalizable, and future studies should further investigate the timing of neutrophil infiltration into the brain following this low-level chronic LPS model in additional mice, specifically in models with genetic modifications that will be useful to further probe the relationship between peripheral inflammation, neuroinflammation, and neurodegeneration. Second, we observed some activation of peripheral neutrophils and some higher neutrophil counts in the brain with some of the saline-injected groups. This makes the interpretations of sustained LPS-induced neutrophil infiltration into the brain beyond 4 h challenging. However, it depicts the continued need for control groups when performing injections and studying inflammation, as the injection itself, or simply handling the mice, may be mediators of inflammation [48]. While neutrophil responses in the brain to stress signals have not been well characterized, studies have demonstrated that acute handling stress and social stress result in inflammation in the periphery that is dominated by neutrophils and neutrophil-specific transcriptional changes [49,50,51]. Finally, we are unable to speak to the longevity of neutrophil responses or how these responses compare to acute stimuli or stimuli given over a shorter duration (i.e., less than 4 weeks) based on this pilot. Future studies should investigate sustained neutrophil inflammation beyond 12 h following LPS injections and the potential for impacts weeks to months after injection. Future studies may also examine how this compares to acute responses to LPS and how chronic exposure changes the response with each subsequent injection. This model can also be used to assess interactions between neutrophils and other cell types in the periphery and brain and investigate molecular mechanisms of neutrophil activation models of neurodegeneration and neuroinflammation. This may be particularly important in the context of microglia, given that previous studies have shown that peripheral stimuli induce epigenetic reprogramming of microglia, suggestive of immune memory in the brain [52]. However, this study provides the foundation for an experimental model to induce neutrophil activation in the periphery and the brain with a subclinical peripheral stimulus, which can be used to understand the role of neutrophils in mediating the periphery–neuroinflammation axis in different mouse models of disease.

## 4. Materials and Methods

### 4.1. Animals 

All mice in the study were generations F3 and F4 from the MRI C57BL/6J strain maintained in-house. The in-house lines are refreshed from The Jackson Laboratory periodically to reduce genetic drift. The mice were housed in individually ventilated and air-filtered cages in a super-barrier mouse room. All cages, bedding, water, and enrichment were autoclaved or UV-treated prior to contact with the mice. Mice always had free access to food and water. All mouse cages were only opened in a biological safety cabinet, and all personnel wore autoclaved lab coats and used sterile gloves. LPS and saline mice were co-housed to reduce cage-to-cage bias. No differences in the behavior of the LPS- vs. saline-injected mice were observable at any time point after injections or during the study. In the 8-week study (Figure 1A), 8 females and 11 males received LPS, and 7 females and 8 males received saline. In the subsequent 4-week study (Figure 1C), 6 females and 6 males received LPS, and 6 females and 5 males received saline.

### 4.2. LPS Injections 

Mice were weighed weekly for 0.5 mg/kg by weight calculations. Vaccine-grade LPS from *Escherichia coli* 0111:B4 (InvivoGen, San Diego, CA, USA) or United States Pharmacopeia (USP) sterile-grade saline (z1376, Cytiva, Marlborough, MA, USA) were 0.22 uM sterile filtered prior to injections. Mice were injected IP with LPS or sterile saline once a week for 8 weeks for the preliminary study (Figure 1A) and once a week for 4 weeks for the next iteration (Figure 1C).

### 4.3. Blood and Brain Collection

Baseline blood was collected the day before the first injection. In the initial experiment, submandibular blood was collected 12 h after the 4-week and 8-week (final) LPS or saline injection. For the next iteration of the study, submandibular blood was collected at 4, 8, or 12 h after the 4-week (final) LPS or saline injection just prior to euthanasia and brain collection. Following the submandibular bleeds, deep anesthetization was carried out with avertin via IP injection. A cardiac puncture was then performed to obtain additional blood prior to whole body perfusion with 20 mL of PBS to flush the vasculature (until fluids ran clear). Success of the perfusion was determined based on coloration before proceeding as previously described [53]. Following perfusion, the brain was then removed and placed in R10 (10% fetal bovine serum in RPMI 1640 with L-glutamate and 25 mM HEPES) and kept on ice until tissue processing. 

### 4.4. Brain Tissue Processing

One hemibrain was enzymatically digested with media (RPMI 1640 with L-glutamate and 25 mM HEPES) supplemented with Liberase (40 µg/ mL, Sigma-Aldrich, St. Louis, MO, USA) and DNAse (4 µg/mL, Sigma-Aldrich, St. Louis, MO, USA) for 45 min at 37 C with vigorous stirring and then ground through a 70 µm cell strainer, as previously described [54]. Isolated brain leukocytes were separated via a percoll gradient as previously described [55] for flow cytometry analysis. The second hemibrain was fixed in 10% buffered formalin for 24 h and then transferred to 70% ethanol until the paraffin-embedding procedure for microscopy staining. 

### 4.5. Staining of Blood and Brain Tissue for Flow Cytometry

Plasma was removed from whole blood and frozen for cytokine analysis, and the volume removed was replaced with 1X phosphate buffered saline (PBS). Next, 25 µL of plasma-removed blood and was lysed for 10 min with 1× Red Blood Cell (RBC) Lysis Buffer (10× RBC, 420302, Biolegend, San Diego, CA, USA). All blood and brain samples were washed twice with 1× PBS and incubated for 5 min with a fixable viability stain (L34988, Invitrogen, San Diego, CA, USA). Samples were then incubated with a Fc block (TruStain FcX, 101320, Biolegend, San Diego, CA, USA) for 5 min. All samples were stained for 20 min with the following anti-mouse antibodies: Ly6G Brilliant Violet 421 (127628, Clone 1A8, Isotype Rat IgG2b, k, Biolegend, San Diego, CA, USA), CD45 APC (103112, Clone 30-F11, Isotype Rat IgG2b, k, Biolegend, San Diego, CA, USA), CD62L PE (104408, Clone MEL-14, Isotype Rat IgG2b, k, Biolegend, San Diego, CA, USA), and CD11b (101216, Clone M1/70, Isotype Rat IgG2b, k, Biolegend, San Diego, CA, USA). Samples were fixed with 1x fixation buffer for 20 min (420801, Biolegend, San Diego, CA, USA) and then washed twice with 1X PBS with 10% FBS wash (FACS wash). Fluorescence data were collected by flow cytometry on a Sony SH800S cell sorter. Compensation beads were stained with the fluorophore-conjugated antibodies used to stain the blood and brain samples, and compensation matrices were applied across all samples. Fluorescence minus one (FMO) tubes were run on each fluorophore-conjugated antibody used in the study to set positive gates. Unstained sample controls demonstrated consistent low background fluorescence across all channels. FlowJo (version 10.9.0, BD Biosciences, Franklin Lakes, NJ, USA) was used to analyze the data. Cells were gated based on surface marker expression and/or scatter properties (Figure S1) [56]. 

### 4.6. Soluble Analyte Analyses

Cytokine and chemokines involved in neutrophil mobilization and activation were measured using a custom multiplex bead array kit (LEGENDPlex, Biolegend, San Diego, CA, USA). Twelve cytokines were measured in duplicate, including CXCL1, CXCL2, G-CSF, IL-1b, IL-6, IL-4, TGFb, TNFa, GM-CSF, INFg, IL-17A, and IL-10. The assay was performed according to manufacturer’s instructions, collected on an SH800 (Sony), and analyzed using the LEGENDPlex Cloud-Based Data Analysis Software version 2023-02-15 (Biolegend, San Diego, CA, USA and Qognit, San Carlos, CA, USA). Neutrophil elastase was measured via ELISA using a pre-validated kit following manufacturer’s instructions (Abcam, Cambridge, UK) and read using a Mini ELISA Plate Reader (Biolegend, San Diego, CA, USA).

### 4.7. Immunohistochemistry 

Formalin-fixed paraffin-embedded sections (5 µm thickness) were baked at 60 °C for 20 min, dewaxed in xylene for 1 h, and rehydrated through an alcohol series. Antigen retrieval with 1X Reveal Decloaker (Biocare Medical, Pacheco, CA, USA) was performed using the capillary gapping method with a steamer for 35 min. Once sections were cooled and washed, they were blocked in Background Sniper (Biocare Medical, Pacheco, CA, USA) for 10 min. Primary antibodies were diluted in Background Sniper as follows and added to sections overnight at 4 °C: Chicken anti-Mouse/Human/Rat GFAP (PA1-10004, Invitrogen, San Diego, CA, USA) and Rabbit anti-human/mouse Recombinant Anti-S100A8 (ab92331, Abcam, Cambridge, UK) were diluted to 1µL/mL, and Goat anti-Human/Mouse MPO (AF3667, R&D, Minneapolis, MN, USA) was diluted to 0.5 µL/mL. Sections were washed in TBS with 0.1% Triton X (TBST) twice and incubated in species-specific secondary antibodies in the following dilutions for 2 h at room temperature: Donkey anti-Chicken Ax594 at a 2 µL/mL, Donkey anti-Rabbit Ax594 at 1 µL/mL, and Donkey anti-Goat IgG Ax488 at a 4 µL/mL. Slides were washed in TBST and distilled water, and then coverslips were mounted onto sections with Vectashield HardSet Antifade Mounting Medium with DAPI (Vector Laboratories, Newark, CA, USA) before imaging. Sections were imaged at 20× and 40× on an Olympus Fluoview FV1000, and neutrophils were counted on a Zeiss Axio Imager.M1 (20× and 40× objective).

### 4.8. Power Calculations and Statistical Analyses

This study was designed to have 0.81 power to detect a 20% difference in means with a standard deviation of 10% in neutrophils in the periphery between saline and LPS groups at each timepoint with an alpha level of 0.05 (n = 4 per timepoint). Statistical significance was assessed between the LPS and saline groups across timepoints by 2-way ANOVA followed by post hoc assessments within each timepoint using Tukey’s multiple comparisons test. 

## Data Availability

The datasets generated and analyzed during this study are available from the corresponding author upon reasonable request.

## Supplementary Materials

The following supporting information can be downloaded at: https://www.mdpi.com/article/10.3390/ijms25105357/s1.
