# Supplementary material for: A Pilot Study to Investigate Peripheral Low-Level Chronic LPS Injection as a Model of Neutrophil Activation in the Periphery and Brain in Mice
Source: Int J Mol Sci. 2024 May 14;25(10):5357. doi: 10.3390/ijms25105357 (PMC11120811; doi:10.3390/ijms25105357)
Supplement: Supplementary file 1 [file ijms-25-05357-s001.zip › ijms-2926463-supplementary.pdf]

Supplementary Information

Peripheral Low Level Chronic LPS Injection as a Model of Neutrophil Activation in the Periphery and Brain in Mice

Michelle Aries, Makayla Cook and Tiffany Hensley-McBain

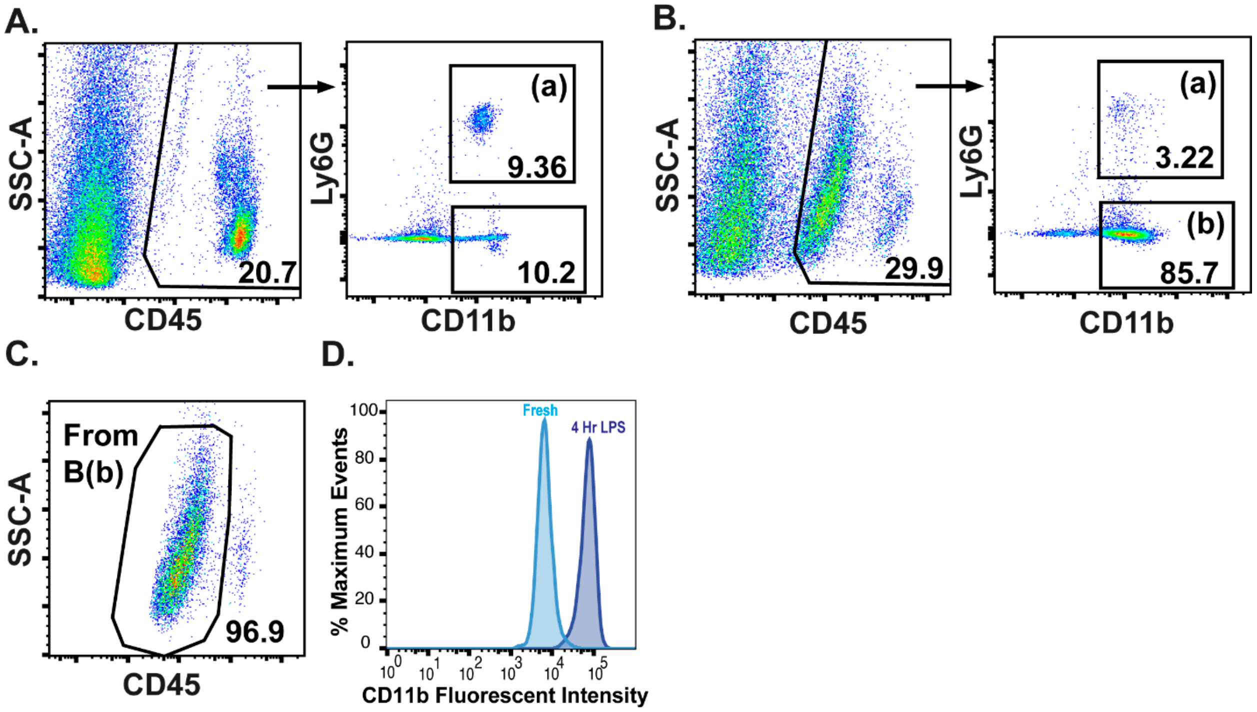

Figure S1. Identification of neutrophils in blood and brain tissue. Example gating strategy in C57BL/6J mouse blood (**A**) and brain tissue following saline injection (**B**). (**A**,**B**) Samples are first gated on total cells, singlets (FSC-A vs. FSC-H), and live cells. Neutrophils are identified as CD45+CD11b+Ly6G+ (a). (**C**) Microglia are identified as CD11b+Ly6G- and CD45-intermediate. (**D**) Shift in CD11b median fluorescent intensity on blood neutrophils following LPS exposure.

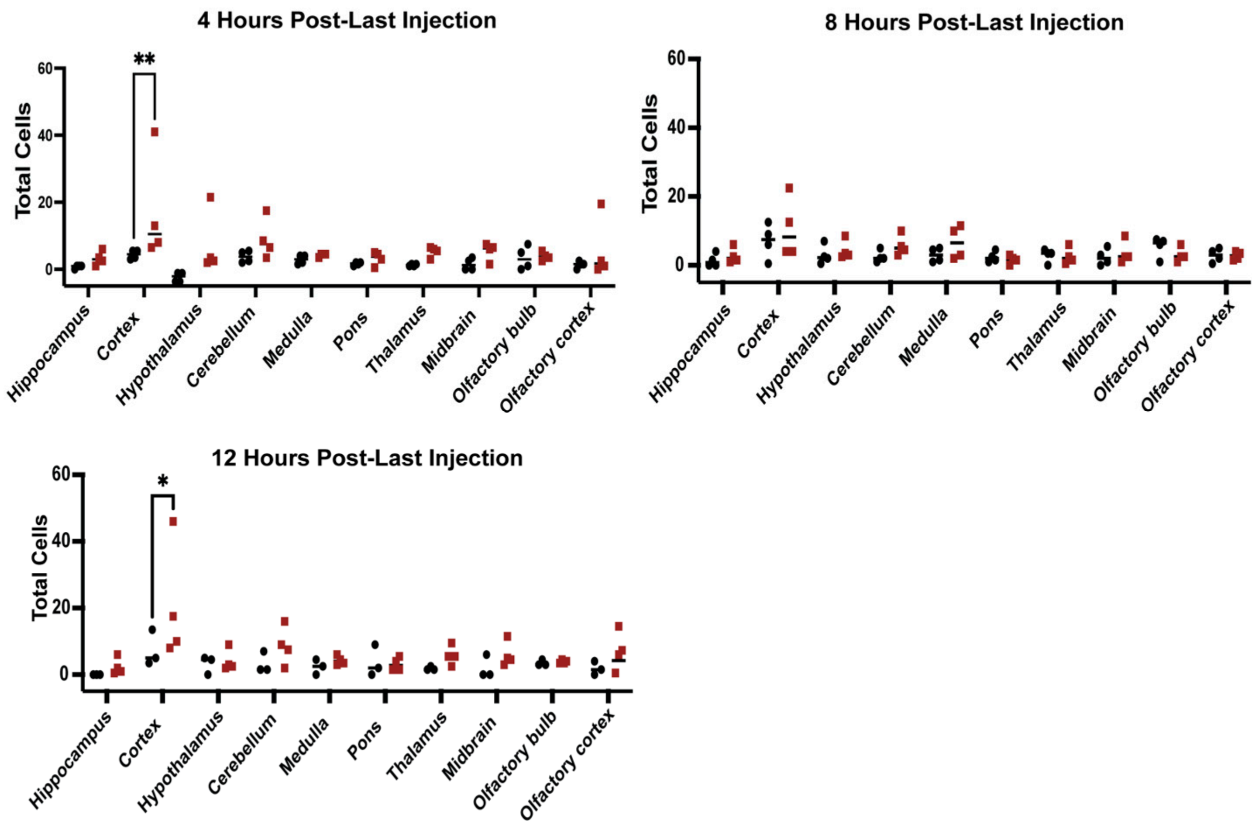

Figure S2. Neutrophils in mouse brain regions following LPS and saline injections. Neutrophils counted via anti-MPO staining and given as a total within the brain region. Each datapoint represents an average count from 2 sagittal sections. Statistical significance was assessed between the LPS and saline groups within the same hour post-last injection by 2way ANOVA followed by Tukey's multiple comparisons test with multiplicity adjusted P values represented as * $p < 0.05$, ** $p < 0.01$.

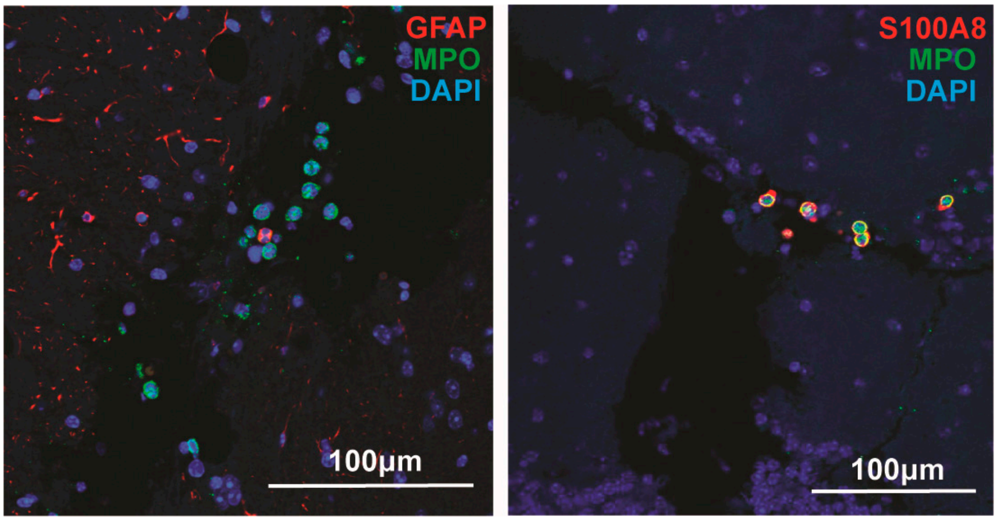

Figure S3. MPO staining colocalizes with **S100A8**, a protein highly expressed in neutrophils, and stains for cells with multilobed nuclei. (**Left**) Mouse brain stained with anti-MPO (green), DAPI (blue), and GFAP (red) demonstrating cells with distinct multilobed nuclei near areas of inflammation in mouse brain tissue (cerebellum 40X). (**Right**) Image of mouse cerebellum (40X) stained with anti-MPO (green), DAPI (blue), and S100A8 (red).

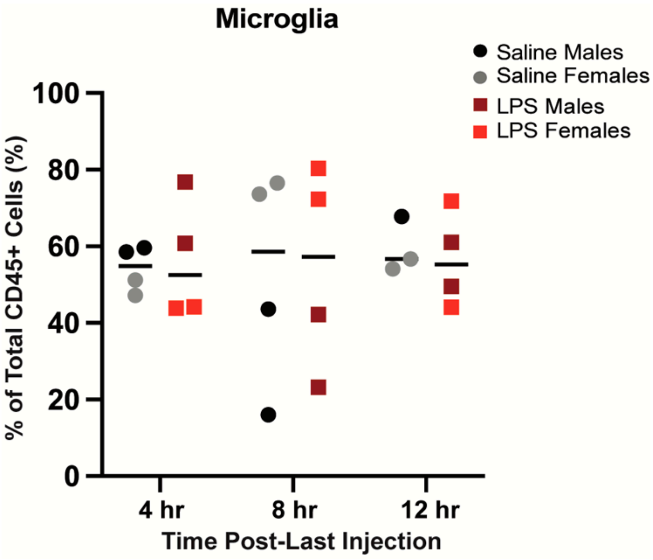

Figure S4. Assessment of microglia frequencies in the brain following chronic low-level LPS injections. Microglia were assessed via flow cytometry and gated as described in Supplementary Fig 1. Statistical significance was assessed between the LPS and saline groups across timepoints by 2-way ANOVA followed by post-hoc assessments within each timepoint using Tukey's multiple comparisons test.
